# Identification of Differentially Expressed Genes and SNPs Linked to *Vibrio mimicus* Resistance in Yellow Catfish (*Pelteobagrus fulvidraco*)

**DOI:** 10.3390/ijms27010441

**Published:** 2025-12-31

**Authors:** Wenjuan Tong, Mengjie Yuan, Songjin Liu, Linwei Yang, Yang Zhou, Qin Tang

**Affiliations:** College of Fisheries, Huazhong Agricultural University, Wuhan 430070, China; tongwenjuan@webmail.hzau.edu.cn (W.T.); ymj0016@gmail.com (M.Y.); 2824542706@webmail.hzau.edu.cn (S.L.); 2023308120057@webmail.hzau.edu.cn (L.Y.); zhouyang@mail.hzau.edu.cn (Y.Z.)

**Keywords:** *Pelteobagrus fulvidraco*, *Vibrio mimicus*, GWAS, SNPs

## Abstract

*Vibrio mimicus* infection poses a severe threat to the sustainable aquaculture of yellow catfish (*Pelteobagrus fulvidraco*), a commercially important freshwater species of the order Siluriformes. To reveal the genetic mechanisms underlying the resistance to this pathogen, we established an infection model and integrated genome-wide association study (GWAS) and transcriptomics to identify key resistance loci and genes. Firstly, from whole-genome re-sequencing (WGRS) and high-quality genotypic data, six SNP loci significantly associated with resistance to *V. mimicus* were identified, which were annotated to 17 immune-related candidate genes. Notably, the *rac2* gene associated with the locus Chr15:3,227,652 exhibited significantly differential expression in skin tissue. Through transcriptomic analysis, 6684 and 6616 differentially expressed genes were identified from the skin and muscle tissues, respectively. Functional enrichment analysis revealed that the skin, as the first line of defense against pathogens, prioritizes the activation of immune defense mechanisms, whereas muscle tissue responds to infection-induced stress primarily by regulating metabolic processes. Quantitative real-time PCR (qRT-PCR) validated that *rac2* enhances the antibacterial capacity of yellow catfish in skin tissue by regulating the expression of NADPH oxidase complex subunits *ncf1* and *ncf4*. This study reveals, for the first time, the core functional genes of yellow catfish associated with resistance to *V. mimicus* infection, providing theoretical support for disease-resistant breeding of this species.

## 1. Introduction

Yellow catfish is rich in nutrition and well-adapted to artificial feed as well as intensive aquaculture environments. However, under high-density farming conditions, its sustainable development is threatened by frequent outbreaks of bacterial diseases. Notably, skin ulcers and rot induced by *V. mimicus* are particularly severe, leading to mortality rates of 80–100% [1]. In addition, the clinical symptoms include muscle necrosis and internal hemorrhage, which exert an extremely severe impact on both aquaculture production and economic benefits [2,3,4]. The pathogenicity of *V. mimicus* is closely associated with its diverse virulence factors, which include outer membrane protein OmpU, hemolysins, enterotoxins, and iron acquisition systems [3,5]. While environmental factors such as pH and salinity, combined with the quorum sensing mechanism of bacteria, exacerbate the outbreak of such diseases [6,7,8]. Currently, the lack of commercial vaccines, and the use of antibiotics is restricted by issues of drug resistance as well as regulatory constraints. Therefore, disease prevention and control has become a major challenge in the yellow catfish aquaculture industry.

In recent years, research on *V. mimicus* infection in fish has made certain progress. For instance, in yellow catfish, researchers have used genomic analysis to reveal the genetic characteristics of *V. mimicus* and its pathogenesis-related mechanisms. Whole-genome sequencing of the *V. mimicus* strain SCCF01 has unveiled multiple genes associated with adhesion, toxin production, and secretion systems [2,9]. Furthermore, specific molecular regulatory pathways have been investigated. In yellow catfish, *V. mimicus* activates the immune pathways and leads to a significant upregulation of immune-related genes such as *il-1*, *il-6*, and *mmp-9* in the skin and muscle tissues, accompanied by apoptosis and inflammatory responses [4]. In grass carp (*Ctenopharyngodon idella*), the immune response mechanism following *V. mimicus* infection has also been studied. It was found that interleukin-1β (*il-1β*) can enhance the expression of antimicrobial peptides by activating the NF-κB pathway, thereby resisting *V. mimicus* invasion [10]. Furthermore, in studies on Cyprinid fish such as crucian carp (*Carassius auratus*), an oral recombinant lactobacillus vaccine expressing the *V. mimicus* OmpK protein has been shown to reduce infection mortality by inducing a mucosal immune response [11]. However, the genetic mechanisms underlying yellow catfish resistance to *V. mimicus* remain unclear. The lack of molecular markers associated with disease resistance traits leads to low efficiency in traditional selective breeding and slow progress in the development of disease-resistant elite varieties. Therefore, systematic genetic studies are urgently needed to identify key disease-resistant genes and molecular markers, thereby providing theoretical support for the formulation of efficient disease-resistant breeding strategies.

In recent years, molecular marker-assisted breeding (MAS) has accelerated the breeding process and improved selection efficiency in aquaculture [12]. Traditional markers such as Restriction Fragment Length Polymorphism (RFLP), Random Amplified Polymorphic DNA (RAPD) [13], Amplified Fragment Length Polymorphism (AFLP) [14], and Simple Sequence Repeat (SSR) [15] have been commonly utilized. Currently, Single-Nucleotide Polymorphism (SNP) has gained prominence in molecular marker research due to its widespread genomic distribution, low mutation rate, and high detection throughput [16]. SNP markers have been successfully applied in the selective breeding of various economically important fish species. In a study on common carp (*Cyprinus carpio*), a GWAS analysis identified 39 growth-related SNP markers, with significant associations found for the genes *tox*, *plk2*, and *cd163* [17]. In Atlantic salmon (*Salmo salar*), SNP loci associated with growth and sexual maturation were screened using a 6.5K SNP array. It was found that the genomic markers *ssa10*, *ssa02*, *ssa13*, *ssa25*, and *ssa12* showed significant correlation with growth performance [18]. In the breeding of Nile tilapia (*Oreochromis niloticus*), researchers developed a 65K SNP liquid array, which was then used to identify Qualitative Trait Loci (QTL) regions on chromosomes 7 and 5 that show significant associations with weight gain and feed intake, respectively [19]. Furthermore, SNPs have been widely applied in the disease resistance analysis of various economically important fish species. For instance, in mandarin fish (*Siniperca chuatsi*), *il-6*-related SNP markers were linked to resistance against infectious spleen and kidney necrosis virus (ISKNV) [20]. In Atlantic salmon (*Salmo Salar*), a combination of Restriction site-Associated DNA Sequencing (RAD-Seq) and QTL mapping identified a QTL and 50 SNP loci associated with resistance to infectious pancreatic necrosis (IPN) [21]. In half-smooth tongue sole (*Cynoglossus semilaevis*), 33 molecular loci significantly associated with disease-resistant traits were screened out using GWAS, population genetic differentiation index, and nucleotide diversity analysis [22]. In large yellow croaker (*Larimichthys crocea*), researchers conducted a study on resistance to visceral white nodule disease. Using a genome-wide association study, they identified 13 disease resistance-related genes and three key regions. Combined with transcriptome analysis, the study further revealed the important role of the *cd82a* gene in the p53 signaling pathway in resistance regulation [23]. These findings provide molecular targets for fish disease-resistant breeding. In addition, the integration of multi-omics data has become a new trend in the systematic analysis of disease resistance mechanisms [24,25]. For example, integrative analysis of GWAS and RNA-seq can effectively narrow down the range of candidate genes, improve the accuracy of functional gene mining, provide a more comprehensive perspective for analyzing the complete mechanism from genetic variation to phenotypic regulation, and promote the development of disease-resistant breeding in aquatic animals toward precision and high efficiency.

In this study, a reliable *V. mimicus* infection model was successfully established. Disease resistance differences among different individuals were identified through histopathological, biochemical and survival analyses. GWAS was carried out to identify significant SNPs associated with disease resistance. And RNA-seq data were integrated to identify the associations between SNP loci and candidate genes. This study provides crucial theoretical support for the disease-resistant genetic breeding of yellow catfish. It not only offers potential molecular markers for selective breeding but also enhances the understanding of the immune defense mechanisms of this species under natural attack. Based on this research, it is expected to screen out elite individuals and significantly enhance the disease resistance of yellow catfish populations, thereby reducing economic losses.

## 2. Results

### 2.1. Strain Identification, Determination of LC_50_, and Establishment of Infection Models

**Figure 1:**
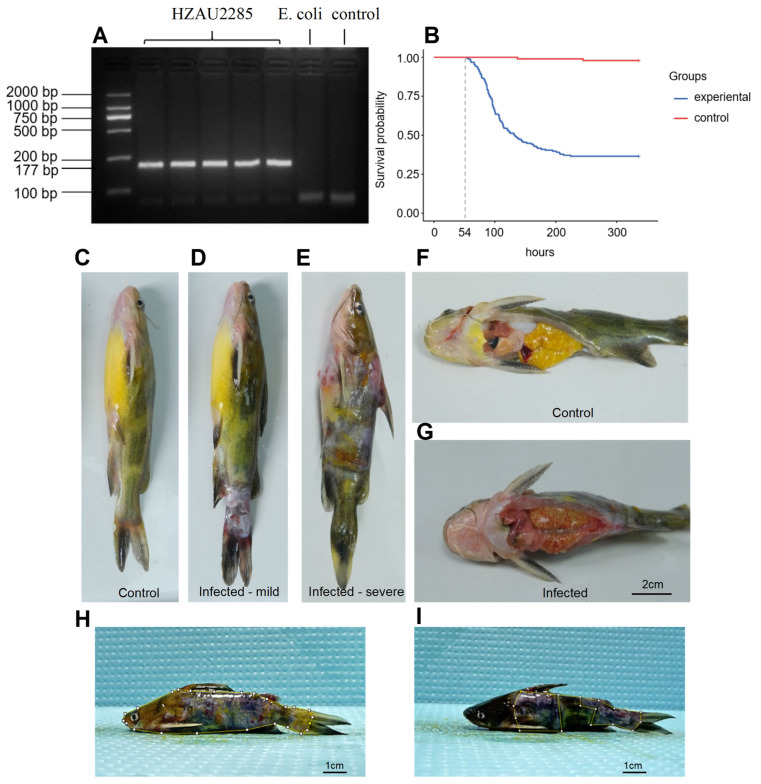
Identification of *V. mimicus* strains and establishment of the yellow catfish infection model. (**A**) Identification of *V. mimicus* through duplex PCR targeting the dnaJ gene; (**B**) survival curves of yellow catfish after *V. mimicus* infection; (**C**) morphological observations revealed that the control group exhibited an intact body surface; (**D**) the fish body exhibited mild decay, with partial ulcers and associated tissue necrosis after *V. mimicus* challenge; (**E**) the fish body exhibited severe decay, extensive tissue damage, and deep lesions after *V. mimicus* challenge. (**F**,**G**) A comparative analysis of anatomical structures between the control and infected groups revealed that the control group exhibited well-organized organ structures without pathological alterations, while the infected individuals showed gallbladder rupture and hemorrhagic lesions in the liver, spleen, kidney, and ovary. (**H**,**I**) The measurement of lateral and ventral lesion areas of yellow catfish using ImageJ (v1.54f).

In this investigation, the strain was initially identified as *V. mimicus* utilizing duplex PCR targeting the *dnaJ* gene. Electrophoresis analysis revealed a band at 177 bp, while no band was evident at 375 bp, thus confirming the strain as *V. mimicus* rather than *V. cholerae*. Subsequent BLAST (https://blast.ncbi.nlm.nih.gov/Blast.cgi, accessed on 25 March 2024) alignment on NCBI corroborated this identification (Figure 1A). A standard curve correlating bacterial concentration with absorbance was established through plate-counting and measurement of optical density, yielding a linear regression equation of *y* = 1.0 × 10^8^*x* + 2.0 × 10^6^ (*R*^2^ = 0.99) (*x* represents bacterial concentration, and *y* represents absorbance) (Supplementary Figure S1A). Then gradient-diluted bacterial suspensions were prepared to infect yellow catfish (Supplementary Figure S1B). Subsequently, The LC_50_ was calculated as 1.78 × 10^5^ CFU/mL by Spearman–Karber method. Using this concentration, a pathological model of yellow catfish infected with *V. mimicus* was successfully established via natural immersion. The results indicated the initial mortality rate in the infected group at 54 h post-infection (hpi), with peak mortality observed between 96 and 120 hpi (*p* = 0.0015) (Figure 1B). It is worth noting that considerable interindividual differences in survival time were observed, reflecting substantial variability in physiological status and genetic diversity within the population. No mortality was observed in the control group throughout the experiment. Post mortem observations of the morphology of the subjects revealed the presence of superficial lesions that were indicative of infection (Figure 1C). Individuals exhibiting mild infection demonstrated heightened pigmentation of the body surface, localized exudation of skin mucus, sporadic bleeding points, and superficial ulcers (Figure 1D). Individuals with severe infection presented with extensive ulcerative lesions, necrotic and shed skin tissue, exposed deep muscle tissue, and diffuse bleeding (Figure 1E). Further anatomical examination revealed that the visceral organs of fish in the infected group all exhibited typical pathological changes, including skin hyperemia at the bases of the pectoral and dorsal fins, vasodilation and hemorrhage in the gill filaments, anal redness and swelling, hepatomegaly (enlarged liver) with petechial hemorrhage, hemorrhagic lesions in the spleen, and gallbladder rupture in some individuals (Figure 1F,G). ImageJ (v1.54f) was used to calculate the lateral and ventral lesion areas, as well as the percentage relative to the total body surface area (Figure 1H,I).

### 2.2. Histopathological Analysis of Yellow Catfish Infected with V. mimicus

**Figure 2:**
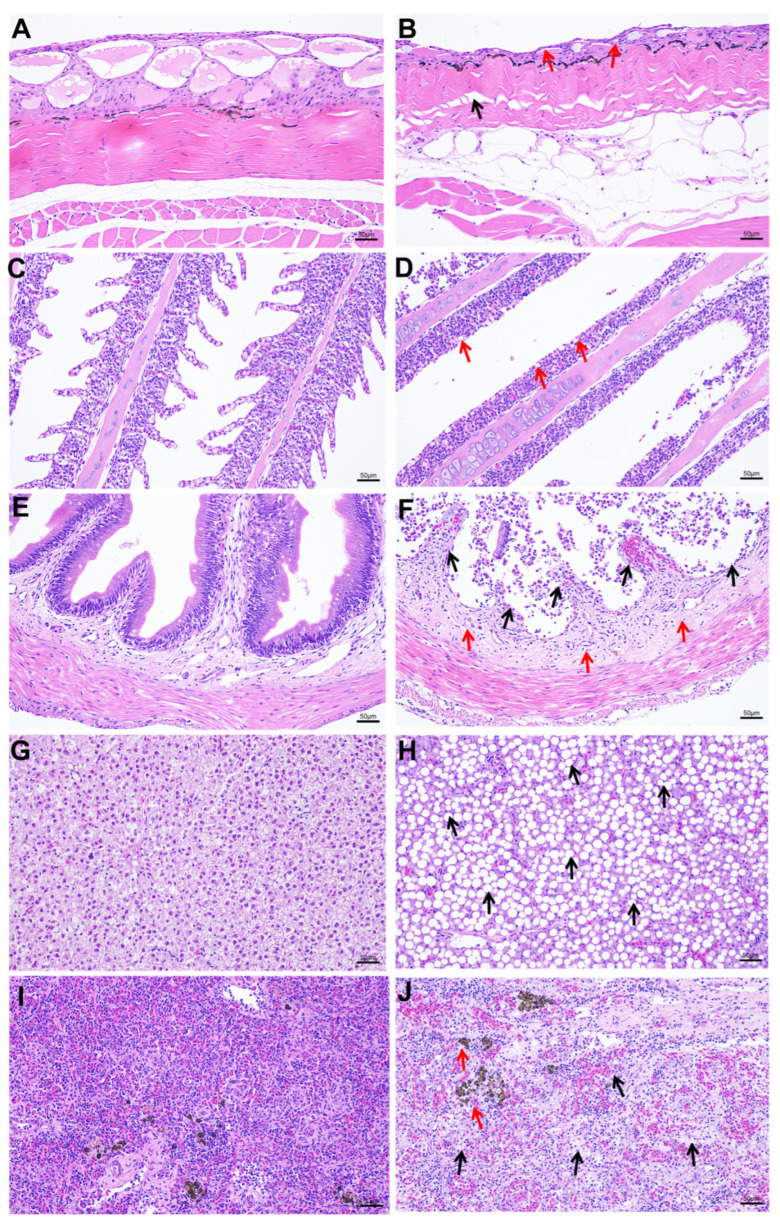
Comparative histopathological characteristics of tissues between control and infected groups. (**A**) Control of skin; (**B**) infected skin, with red arrows indicating reduced mucous cells and black arrows showing loosely arranged collagen fibers; (**C**) control of gill; (**D**) infected gill, with red arrows representing detached lamellae; (**E**) control of intestine; (**F**) infected intestine, with red arrows indicating absence of crypt structures and black arrows showing epithelial shedding in mucosal layer; (**G**) control of liver; (**H**) infected liver, with black arrows indicating steatosis; (**I**) control of spleen; (**J**) infected spleen, with red arrows representing hemosiderin deposits and black arrows showing disorganized splenic nodules with indistinct demarcation between red and white pulp.

Compared to the control group, yellow catfish in the infected group displayed distinctive pathological alterations across multiple organs. Histological examination of the skin revealed evident epidermal atrophy, reduced mucous cell count, localized collagen fiber disarray in the dermis, and muscle fiber dissolution (Figure 2A,B). The interlamellar cell masses in the gill tissue were thinned, and extensive gill lamellae detachment was observed in some gill filaments (Figure 2C,D). The epithelial layer of the intestinal mucosal layer is severely denuded, with the lamina propria exposed and the crypt structure of the mucosal layer lost, but no edema is observed in the submucosal layer (Figure 2E,F). In the liver tissue, most hepatocytes exhibit steatosis, and diffuse round vacuoles are visible in the cytoplasm. The hepatic sinusoids are uniform in size without dilatation, and the hepatic cords are arranged neatly and densely (Figure 2G,H). In the spleen tissue, the structure of splenic nodules is disorganized, the boundary between red and white pulp is indistinct, the number of lymphocytes is reduced and arranged loosely, and a more amount of hemosiderin deposition is locally observed in the tissue than control (Figure 2I,J).

### 2.3. Genome-Wide Association Analysis for Screening Resistance Loci Against V. mimicus Infection

Among the 150 experimental fish examined for GWAS analysis and SNP screening, 66 exhibited severe ulcers, 38 displayed mild ulcers, and 46 were deemed healthy. The mortality rate was 92 fish out of 100, with 58 fish exhibiting survival. A subsequent statistical analysis revealed that there was no significant difference in growth traits between the survival group and the death group (*p* = 0.26 for body length and *p* = 0.06 for body weight, respectively) (Supplementary Table S1).

**Figure 3:**
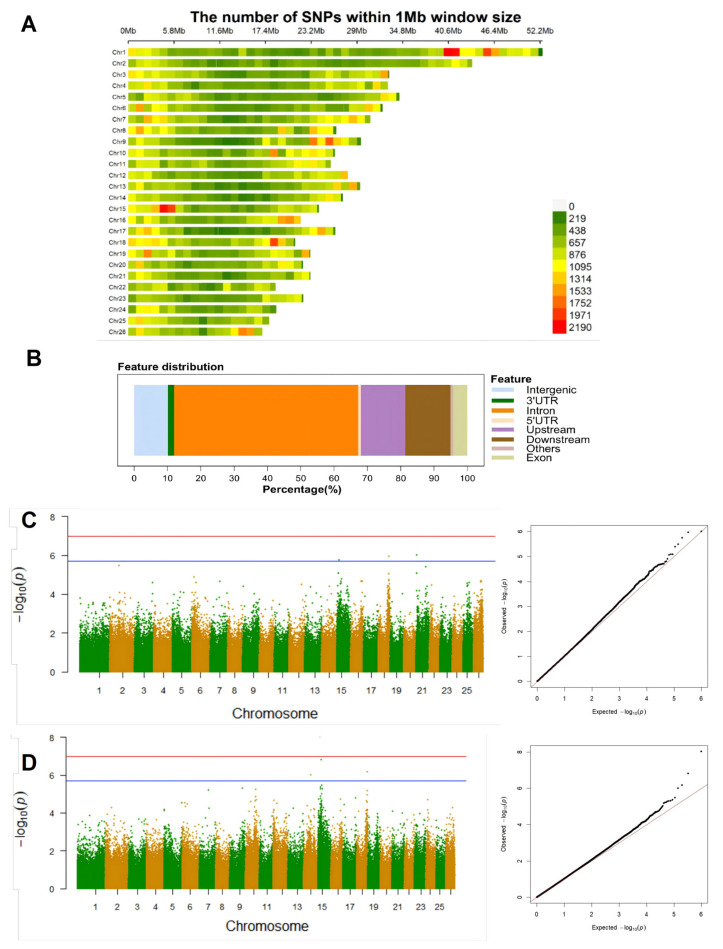
GWAS analysis for screening resistance loci against *V. mimicus* infection. (**A**) Density map showing the genome-wide distribution of SNP loci (by 1 Mb windows); (**B**) percentage histogram of SNP distribution at different genome regions; (**C**) screening of SNP loci associated with survival and mortality via Manhattan plots, the green and yellow blocks represent different chromosomes, with significance threshold set at 1.02 × 10^−7^ and suggestive threshold at 2.04 × 10^−6^; (**D**) screening of SNP loci associated with lesion severity via Manhattan plots, with the same thresholds as in figure C, points deviating further from the diagonal line indicate significant associations or biases. Red line: the threshold; Blue line: the suggestive threshold.

Whole-genome resequencing was employed to obtain a total of 1263.80 GB of high-quality raw reads. The data quality assessment revealed that the average values of Q20 and Q30 across all samples reached 95.18% and 89.62%, respectively, suggesting the presence of adequate sequencing data quality. For each sample, the genome alignment rate was above 98.75%. Subsequent to genotyping and stringent filtration, 490,395 high-quality SNP loci were identified. The genome-wide distribution of SNP loci is shown in Figure 3A. Chromosomes 1, 9, 15, and 18 have been identified as regions with notable SNP enrichment, suggesting the potential presence of significant information concerning *V. mimicus* resistance within these regions. Annotation of the SNP locus revealed that 92.33% of the SNP loci are located in non-coding regions (Figure 3B), among which the intronic regions account for 55.26%, the upstream and downstream regulatory regions of genes account for 26.95%, and the intergenic regions account for 10.12%. Only 4.22% of the SNP loci are located in exonic regions, with the 3′UTR and 5′UTR accounting for 1.86% and 0.75%, respectively.

Six significant SNP loci were evaluated using the mixed Linear model (MLM), with a significance threshold set at 1.02 × 10^−7^ (*p* = 0.05/N, N representing the total number of SNPs) and a suggestive threshold at 2.04 × 10^−6^ (*p* = 1/N), where N denotes the total number of SNPs. The analysis was performed using lesion severity (0: normal; 1: mild; 2: severe) and survival/mortality (0/1) as phenotypes. Notably, three SNP loci surpassed the suggestive threshold for the lesion trait, especially at Chr15:3,227,652, Chr18:18,167,796, and Chr21:650,247 (Figure 3C). In contrast, for the survival/mortality trait, four SNP loci exceeded the suggestive threshold, including Chr14:13,668,655, Chr15:3,227,652, Chr15:4,953,448, and Chr18:19,797,963 (Figure 3D). Remarkably, Chr15:3,227,652 exhibited significant associations with both traits, suggesting a pivotal role in the immune response of yellow catfish. Of the six loci significantly associated with both traits, four are located in intronic regions, while the other two are in intergenic regions. By scanning the 500 kb range upstream and downstream of these loci, a total of 349 potential candidate genes were identified. These genes are mainly involved in processes such as lipid metabolism, the MAPK signaling pathway, phagocytosis, immunoglobulin receptor binding, and B cell activation. After further investigation into the functions of these genes, 17 immune-related genes were finally obtained (Table 1).

### 2.4. Screening of V. mimicus Resistance Genes by Transcriptome Differential Analysis

To investigate the regulatory roles of candidate genes during *V. mimicus* infection, we conducted transcriptome sequencing on the skin and muscle tissues of both the control group and the lesion group. A total of 77.63 GB raw reads were generated, with average Q20 and Q30 values exceeding 97.37% and 94.57%, respectively, indicating high sequencing quality. Following filtering, 514,822,082 clean reads were obtained. Genome alignment yielded rates ranging from 64.70% to 85.10% across all samples (Supplementary Table S2). Principal component analysis (PCA) revealed distinct clustering patterns corresponding to tissue type and infection status, with samples within the same group (consistent tissue type and infection status) showing tight aggregation. This observation indicates strong intra-group consistency and validates the robust parallelism of the experimental samples (Supplementary Figure S2).

**Figure 4:**
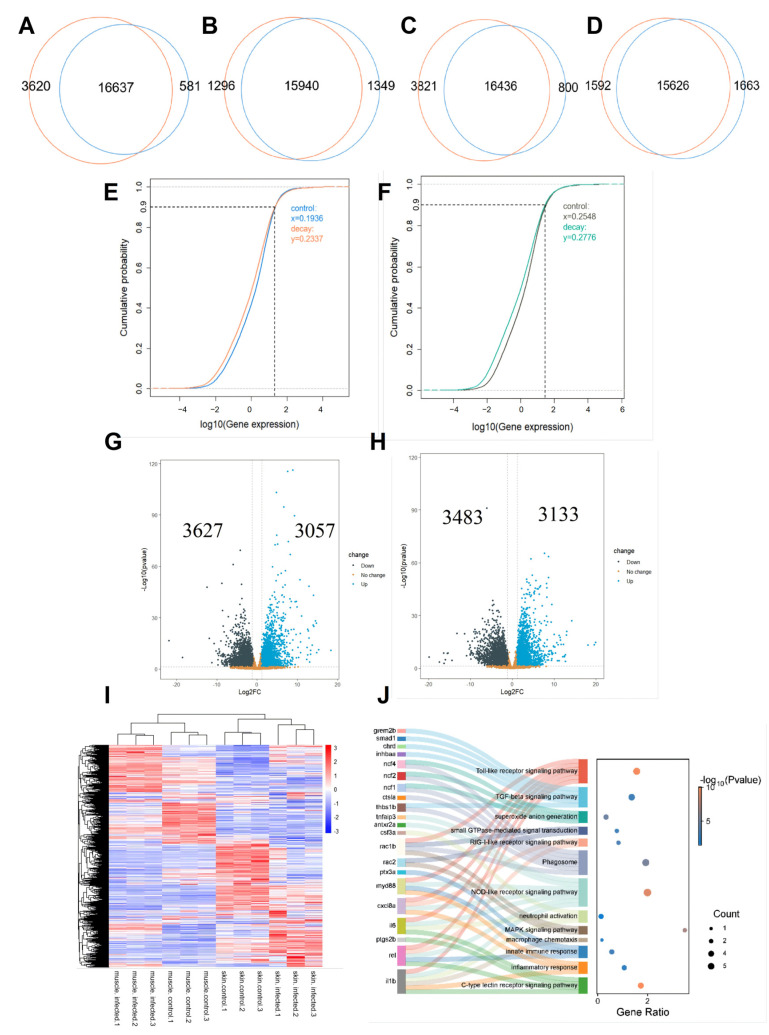
Transcriptomic analysis of the skin and muscle tissues of yellow catfish after infection with *V. mimicus*. (**A**) Venn diagram of expressed genes in control group versus infected group of skin; the orange circles represent the control group, while the blue circles represent the infected group. (**B**) Venn diagram of expressed genes in control group versus infected group of muscle; (**C**) Venn diagram of expressed genes in skin control group versus muscle control group; (**D**) Venn diagram of expressed genes in infected skin group versus infected muscle group; (**E**) cumulative distribution of gene expression in skin, the blue curve represents the control group, and the orange curve represents the infected group; (**F**) cumulative distribution of gene expression in muscle, the gray curve represents the control group, and the turquoise curve represents the infected group, the left side of the vertical dotted line represents the cumulative distribution of 90% of the genes, and the right side represents the cumulative distribution of the top 10% highly expressed genes; (**G**) volcano plot of DEGs identified from skin; (**H**) volcano plot of DEGs identified from muscle; (**I**) clustering heatmap of differentially expressed genes in skin and muscle; (**J**) Sankey of potential marker genes in the skin and their associated pathways.

In the skin tissue, 20,258 and 17,218 expressed genes were detected in the control and infection groups, respectively, with 16,637 common expressed genes, 3620 specifics to the control group, and 581 specifics to the infected group (Figure 4A). In the muscle tissue, 17,236 and 17,289 genes were detected to be expressed in the control and infected groups, respectively, with 15,940 genes expressed in both groups, 1296 specifically expressed in the control group, and 1349 specifically expressed in the skin infection group (Figure 4B). Compared to the muscle control group, the skin control group had more specific expressed genes (Figure 4C), while the number of specific expressed genes in the infection group of the skin was lower than that in the muscle group (Figure 4D). Through cumulative distribution analysis of the expressed genes in the skin and muscle tissues, we found that the expression levels in both tissue groups exhibited a similar distribution pattern. In the skin control group, 90% of gene expression levels were below 0.1936 (log_10_(FPKM)), and in the infection group, below 0.2337 (Figure 4E). In the muscle control group, 90% of gene expression levels were below 0.2548, and in the infection group, below 0.2776 (Figure 4F). This indicates that the overall gene expression levels in the infection group are slightly higher than those in the control group for both skin and muscle tissues. Further differential expression analysis revealed 3627 upregulated genes and 3057 downregulated genes in the skin, and 3483 upregulated genes and 3133 downregulated genes in the muscle (Figure 4G,H). Cluster analysis showed that the differentially expressed genes in skin and muscle tissues exhibited distinct tissue-specific expression patterns, allowing all samples to be accurately distinguished based on their tissue origin (Figure 4I).

Functional analysis of differentially expressed genes reveals that in skin tissue, upregulated genes are primarily enriched in pathways related to neutrophil activation, phagocytosis, autophagy, apoptosis, inflammation, and innate immune response, including macrophage chemotaxis, apoptosis, Toll-like receptor signaling, TGF-β signaling, protein ubiquitination, inflammatory responses, and C-type lectin receptor signaling pathways (Supplementary Figure S3A). Downregulated genes are mainly enriched in fundamental metabolic pathways, including fatty acid metabolism, cell cycle, DNA replication, and wound healing (Supplementary Figure S3B). In addition, a number of several cross-linked pathways including innate immune response, phagosome, neutrophil activation, phagocytosis, and inflammatory response, with highly expressed genes including *rac2*, *ncf1*, *ncf2*, *ncf4*, *il6*, *il1b*, *myd88*, *cxcl8a*, and *rel* identified from the skin. (Figure 4J). These genes could potentially serve as biomarkers for skin immune activation following infection with *V. mimicus* in yellow catfish. In muscle tissue, upregulated genes are mainly enriched in pathways associated with proteasome activity, protein ubiquitination, autophagy, and amino acid transmembrane transport, including RIG-I-like, NOD-like, Toll-like receptor signaling pathways, NF-κB, and cytokine signaling pathways (Supplementary Figure S3C). Downregulated genes are primarily enriched in metabolic pathways such as the TCA cycle, glycolysis, oxidative phosphorylation, and fatty acid β-oxidation (Supplementary Figure S3D). These results indicate that under *V. mimicus* infection, skin serves as an immune barrier, prioritizing the maintenance of defense functions by activating innate immunity and programmed cell death. Conversely, muscle tissue responds to infection by enhancing the synthesis of immune-related proteins. Therefore, subsequent analyses will exclusively integrate the differential genes from skin tissue with GWAS results.

### 2.5. Population Validation of SNP Loci and Verification of Resistance Genes and SNPs Associated with V. mimicus Infection in Yellow Catfish

**Figure 5:**
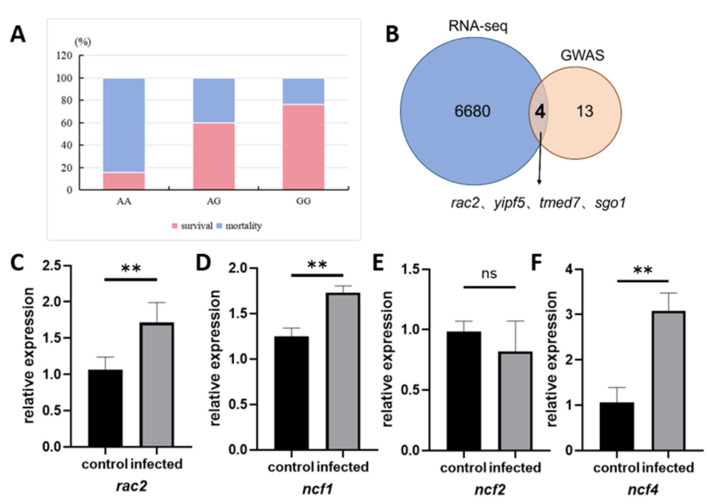
Population validation of SNP loci and verification of resistance genes and SNPs associated with *V. mimicus* infection. (**A**) Population validation of survival rate of different genotypes (AA, AG, GG) at locus Chr15:3227652; (**B**) the Venn diagram of GWAS candidate genes and DEGs from RNA-seq; (**C**–**F**) changes in expression levels of *rac2* and its downstream genes *ncf1*, *ncf2* and *ncf4*, statistical significance was determined at ** *p* < 0.01, with *p* > 0.05 considered non-significant (ns).

To verify whether the polymorphism at the Chr15:3,227,652 locus is associated with disease resistance or survival rate, a *V. mimicus* infection experiment was conducted again in the WT population of yellow catfish. The survival and mortality statuses of individual fish were recorded, and their genotypes at the target locus were determined via Sanger sequencing. The validation population comprised 19 individuals with the AA genotype, 20 with AG, and 21 with GG. The survival rates of AA, AG, and GG-genotyped individuals were 15.79%, 60.00%, and 76.19%, respectively (Figure 5A). Notably, the GG genotype demonstrated a notably higher survival rate, which was consistent with the findings from the previous GWAS.

To further analyze the molecular markers and regulatory mechanism, we integrated 17 immune-related genes obtained from the annotation of significant SNP loci in GWAS analysis with 6684 DEGs identified from skin. Ultimately, *rac2*, *yipf5*, *tmed7*, and *sgo1* were screened to be the key genes (Figure 5B). Subsequent research endeavors focused on the identification of the pathways in which these genes are situated. The results of this investigation demonstrated that *yipf5*, *tmed7*, and *sgo1* are mainly enriched in skin in biological processes, including intracellular protein transport and cell cycle. In contrast, *rac2* is enriched in immune-related pathways including signal transduction, the MAPK signaling pathway, actin cytoskeleton, and focal adhesion. Moreover, existing studies have indicated that *rac2* mediates host defense responses by regulating neutrophil chemotaxis and phagocytosis [26,27]. Therefore, *rac2* was identified as a key candidate functional gene for yellow catfish to resist *V. mimicus* infection.

*rac2* have been shown to regulate *ncf1*, *ncf2*, and *ncf4*, thereby affecting the immune defense capability of neutrophils [28]. To further explore the mechanism of *rac2* gene in immunity, qRT-PCR was used to verify the expression levels of the *rac2* gene in skin and its downstream regulatory genes. Results indicate that the expression levels of *rac2*, *ncf1*, and *ncf4* in the infected group were significantly increased, while the expression level of *ncf2* showed no obvious change (Figure 5C,F). This suggests that *rac2* potentially enhance the host’s antibacterial ability by selectively regulating the expression of NADPH oxidase complex subunits *ncf1* and *ncf4*, thereby activate the oxidative burst response in neutrophils.

## 3. Discussion

*V. mimicus* has become a major pathogenic microorganism threatening the aquaculture industry due to its strong pathogenicity and high lethality. When infecting hosts, most *V. mimicus* strains mainly colonize mucosal tissues such as the host’s skin, gills, and intestines, causing significant pathological damage to these immune barrier organs. To explore its pathogenic mechanism in depth, this study successfully established a pathological model of yellow catfish infected with *V. mimicus* by simulating natural infection, and conducted phenotypic, survival, and histopathological analysis. Among the findings, the multi-tissue pathological damage and enzyme activity changes observed in yellow catfish after *V. mimicus* infection revealed the complex pathogenic mechanism of this pathogen, including systemic invasion of the host and dysregulation of immune metabolism.

A close association has been demonstrated between impairment of mucosal barrier function and pathogen invasion. Following infection by *V. mimicus*, yellow catfish exhibited substantial mucosal structural abnormalities in the skin, gills, and intestines, indicating that the pathogen invades by disrupting the host’s mucosal barrier defense system. Specifically, the reduced number, atrophy of mucus-secreting cells, and local loose arrangement of collagen fibers in the dermis of the skin suggested that *V. mimicus* may enhance its pathogenicity by directly damaging the skin barrier. As important components of mucosal immunity, the atrophy of mucus-secreting cells may reduce the secretion of core immune effector molecules (e.g., antimicrobial peptides and lectins) in mucus, thereby weakening the host’s first line of immune defense [29]. Pathological changes in the gills also reflected the failure of mucosal immune responses. Studies have shown that immunoglobulins (e.g., IgT) and pattern recognition receptors (e.g., TLR5, TLR9) in the gills play key roles in resisting pathogen invasion [30,31]. After bacterial invasion, TLR5 and TLR9 can recognize bacterial flagellin to activate the NF-κB pathway and induce the expression of the pro-inflammatory cytokine TNF-α. The shedding of gill filaments may be related to abnormal immune cell expression or blocked receptor signal transduction [32]. In addition, the destruction of gill filament structure not only impairs respiratory function but also facilitates pathogen invasion into the circulatory system, which is consistent with the high mortality rate often observed in fish after *V. mimicus* infection [33]. The loss of intestinal mechanical barrier integrity was due to epithelial shedding and crypt structure loss in the intestinal mucosal layer. This allowed pathogens and toxins to penetrate the intestinal wall more easily, triggering systemic infection [34]. Notably, pathological changes in the liver and spleen in this study revealed metabolic disorders and immune function damage caused by *V. mimicus*. Hepatic steatosis and cytoplasmic vacuolation in hepatocytes may be related to pathogen toxins interfering with lipid metabolism or mitochondrial function [35]. Although no expansion of hepatic sinusoids was observed, steatosis can reduce the liver’s detoxification capacity, further exacerbating toxin accumulation in the body [36]. The reduction in lymphocyte count and blurred boundary between red and white pulp in the spleen suggested that *V. mimicus* infection may induce immunosuppression by directly killing immune cells or inhibiting lymphocyte differentiation. Meanwhile, hemosiderin deposition reflected the compensatory enhancement of the spleen’s function in clearing red blood cell debris [37].

GWAS is a technique commonly employed in investigating disease resistance in aquaculture species. It has been successfully used in identifying quantitative trait loci (QTLs) and candidate genes associated with resistance to enteric septicemia (ESC) and columnaris disease in catfish [38,39]. In this study, a total of 490,395 single SNP loci were identified through GWAS analysis. Using a mixed linear model with survival/mortality and lesion degree as traits, 6 SNP loci associated with *V. mimicus* resistance were identified, among which four were located in intronic regions and two in intergenic regions. The results suggested that *V. mimicus* resistance in yellow catfish may be affected by gene expression regulation or non-coding functional elements. Despite their inability to encode proteins directly, intronic regions have been demonstrated to play pivotal roles in gene expression. For instance, intronic regions have been shown to regulate resistance traits indirectly by influencing RNA splicing or mRNA stability [40]. SNPs in intergenic regions, on the other hand, may function by regulating the expression of adjacent genes or influencing chromatin structure. In addition, the locus Chr15:3,227,652 showed significant associations in both association analyses of the two resistance traits and was located in a high-frequency SNP region on chromosome 15. This phenomenon suggests that this SNP locus may be part of a key regulatory hub in the immune regulatory network, involved in regulating multiple resistance-related pathways. Furthermore, the genetic polymorphism exhibited in the high-frequency SNP region may indicate that this genomic segment maintains key functional loci related to resistance under selective pressure. Annotation of the Chr15:3,227,652 locus showed that it is located in the intronic region of *rac2*. The protein encoded by *rac2* is a small GTPase belonging to the Rho family, which is mainly involved in processes such as phagocytosis, inflammation, actin cytoskeleton remodeling, cell migration, and intracellular signal transduction [41]. It plays a key role in immune responses. For example, *rac2* mutations in humans can cause immune deficiency diseases [42]. In this study, the expression of the rac2 gene was significantly upregulated in yellow catfish following *V. mimicus* infection, indicating its potential involvement in the species’ immune response. This is consistent with the research result that *rac2* gene expression is significantly upregulated in fruit fly (*Drosophila melanogaster*) upon pathogen infection [43]. Notably, the *rac2* gene was upregulated in both skin and muscle tissues, but its expression level was more significant in the skin. This tissue-specific difference may reflect distinct responses of the two tissues to *V. mimicus* infection. The skin, as the first immune barrier, activates immune defense mechanisms preferentially, while the muscle tissue responds to infection stress by altering metabolism.

Although this study provides a breakthrough for the screening of *V. mimicus*-resistant yellow catfish, it still has certain limitations. For instance, the sample size does not provide a complete representation of the overall genetic diversity of the population, which impedes the detection of rare variants. And the functional verification of candidate genes requires further investigation, for example, gene knockout or overexpression experiments should be explored to clarify the immune functions of significant markers. Furthermore, additional efforts are needed, including the development of immunostimulant based on molecular markers, the establishment of healthy aquaculture models with environment-host synergy optimization, and the research and development of broad-spectrum disease-resistant varieties or combined vaccines targeting *V. mimicus* and other pathogens. These efforts aim to form a systematic integrated disease prevention and control system, providing support for the sustainable development of the yellow catfish industry.

## 4. Materials and Methods

### 4.1. Animals and Bacteria

The yellow catfish used in this experiment were derived from female parents of all-male yellow catfish No. 2, a new aquatic variety (generated by crossing of XX female and YY super-male) developed by the genetic breeding team of Huazhong Agricultural University through a systematic breeding program, which exhibits excellent growth performance. These female parental fish also called all-female fish, were obtained by crossing XX females with XX neo-males (sex-reversed males). At the time of the experiment, the female parents were approximately one year old, with an average body weight of 18.65 g. Healthy female yellow catfish were reared in a recirculating freshwater system (25 ± 2 °C) at the Aquaculture Research Institute of Wuhan Academy of Agricultural Sciences. Fish were fed twice daily with commercial feed (1.5% body weight) and maintained under optimal water quality (daily 1/3 water exchange, with continuous aeration).

*V. mimicus* strain HZAU2285, isolated from diseased yellow catfish in Hubei Province, was provided by the State Key Laboratory of Agricultural Microbiology of China. The HZAU2285 strain was confirmed by duplex PCR using two sets of species-specific primers (Table 2), designed to amplify the *dnaJ* sequences of *V. mimicus* and *Vibrio cholerae*, respectively. Due to the high sequence similarity of the two strains, they share a common upstream primer and use their respective downstream primers. Specifically, the target band amplified by *V. mimicus*-F and *V. cholerae*-R is 375 bp in length, which is used for the identification of *V. cholerae*. The target band amplified by *V. mimicus*-F and *V. mimicus*-R is 177 bp in length, which is used for the identification of *V. mimicus*.

### 4.2. Determination of LC_50_ and Establishment of Infection Model

180 healthy fish (average weight 18.5 ± 3.6 g) were selected and randomly divided into 18 groups, including 3 control groups (*n* = 10) in 0.65% saline solution and 15 experimental groups (n = 10) exposed to serial dilutions of *V. mimicus* strain HZAU2285 (1 × 10^3^ to 1 × 10^7^ CFU/mL) via 1 h immersion.

The immersion process was continuously aerated (dissolved oxygen > 6 mg/L). Water temperature was stabilized at 25 ± 2 °C, with 1/3 daily water exchange and routine water quality monitoring to ensure optimal conditions. Fish were fed commercial diet at 1.5% of body weight twice daily. After immersion, fish were transferred back to aerated freshwater and monitored for 14 days, with daily mortality recorded. The Spearman–Karber method was employed to calculate the lethal concentration 50% (LC_50_) [44].

A total of 300 healthy female yellow catfish (average weight 18.8 ± 6.2 g) were randomly assigned into two groups: the control group (n = 150) immersed in aerated freshwater, and the infected group (n = 150) exposed to *V. mimicus* HZAU2285 at 1.78 × 10^5^ CFU/mL for 1 h to construct the pathological model. The observation period lasted 14 days. The time of death was recorded for each experimental fish upon mortality, and the carcasses were promptly removed. All other conditions were consistent with the LC_50_ determination.

### 4.3. Sample Collection

#### 4.3.1. Histopathological Sample Collection

Three fish were randomly selected from both the control and infected groups, anesthetized with 200 mg/L MS-222, and then dissected to collect skin, muscle, gill, intestine, liver, and spleen tissues. Samples were immediately fixed in 4% paraformaldehyde at 4 °C for 24–48 h, followed by paraffin embedding, sectioning (3 μm), hematoxylin–eosin (HE) staining, and microscopic observation of pathological changes. Samples were immediately fixed in 4% paraformaldehyde (4 °C, 24–48 h), embedded in paraffin, sectioned (3 μm), and stained with hematoxylin and eosin (H&E) for microscopic examination of pathological lesions.

#### 4.3.2. Phenotypic Data Collection

During *V. mimicus* infection, mortality was recorded when fish exhibited loss of equilibrium and cessation of opercular movement for more than 10s. Moribund individuals were immediately anesthetized with 200 mg/L MS-222, followed by measurement of body length and weight. Standardized lateral and ventral photographs of the fish were collected for quantitative analysis of ulcers. Lesion areas and their percentage of the total body surface area were calculated using ImageJ software (v1.53).

#### 4.3.3. DNA Sample Collection

Upon mortality, caudal fins were collected from 150 fish (including phenotypic records), stored in nuclease-free tubes at −20 °C, and processed for genomic DNA extraction using a salting-out protocol. The purified DNA was stored at −20 °C.

#### 4.3.4. RNA Sample Collection

Skin and muscle tissues were harvested from three healthy controls and three infected fish (MS-222 anesthetized) with ulcer areas larger than 20%, preserved in RNase-free tubes, total RNA from yellow catfish samples was extracted by TRIzol (Invitrogen, Carlsbad, CA, USA) according to the manufacturer’s instruction and stored at −80 °C until use.

### 4.4. Genotyping and Genome-Wide Association Analysis

Genomic DNA was extracted from caudal fins subjected to whole-genome resequencing (10× coverage) on the BGI Gene Synthesis Platform (Beijing Biomarker Technologies Co., Ltd., Beijing, China), generating 150 bp paired-end raw reads.

Raw sequencing data were quality-assessed using FastQC (v0.11.5) [45] and filtered with Trim-galore (v0.4.5, https://www.bioinformatics.babraham.ac.uk/projects/trim_galore/, accessed on 25 March 2024) under the following parameters: --clip_R1 8 --clip_R2 8 -q 20 --paired --phred33, high-quality clean reads were used for downstream analysis. Clean reads were aligned to the yellow catfish reference genome HZAU_PFXX_2.0 (NCBI BioProject number PRJNA489116) using BWA (v0.7.17) [46], followed by duplicate removal with Sambamba [47]. Variant calling was performed using GATK (v4.0.12.0) [48] HaplotypeCaller to generate VCF files, which were subsequently hard-filtered with GATK VariantFiltration [48] (QD ≥ 2.0; FS ≤ 60.0; MQ ≥ 40.0; SOR ≤ 3.0; MQRankSum ≥ −12.5; ReadPosRankSum ≥ −8.0; DP < 10.0). Missing genotypes were imputed using Beagle (v4.1) [49], and further SNP filtering was conducted with PLINK (v1.9) [50] to remove markers with call rates below 90%, minor allele frequency (MAF) less than 0.05, or deviation from Hardy–Weinberg equilibrium (*p* < 1 × 10^−4^). Linkage disequilibrium (LD) pruning was performed (window size: 100 kb; step: 1 SNP; r^2^ threshold: 0.5), and SNP density was visualized using the R package CMplot (v4.2.3) with a window size of 1 Mb.

Univariate GWAS for survival/mortality and ulcer severity traits was performed using a mixed linear model (MLM) in GEMMA (v0.98.1) [51]. The computational model is y = Wα + Xβ + μ + e, where y represents the phenotypic value of the yellow catfish’s resistance trait against *V. mimicus*; W denotes the fixed-effects matrix comprising the intercept and covariates; α is the fixed-effects vector used to estimate the influence of genotypes on the phenotype; X is the marker-effects matrix; β is the marker-effects vector; and μ is the polygenic effects vector, following a normal distribution μ ~ N (0, σg^2^K) with mean vector 0 and variance–covariance matrix σg^2^K, where σg^2^ denotes the additive genetic variance and K is the genetic correlation matrix. e is the vector of residuals, following a normal distribution e ~ N (0, σe2I), where σe2 is the variance of non-genetic effects and I is the association matrix. Manhattan and quantile–quantile (Q-Q) plots were generated with the R package qqman (v4.2.3). Genome-wide significance thresholds were determined via Bonferroni correction [52]: α = 0.05/N for significance; α = 1/N for suggestive associations, where N represents the number of independent SNPs. Significant SNPs associated with *V. mimicus* resistance were annotated using SnpEff (v4.3) [53] to identify candidate genes within 100 kb flanking regions, with priority given to immune-related genes for functional validation.

### 4.5. RNA Sequencing and Differential Expression Analysis

Total RNA was extracted from skin and muscle samples were used to construct Illumina libraries and subjected to RNA sequencing on the Illumina Hiseq2500 platform (Illumina, San Diego, CA, USA), generating 150 bp paired-end raw reads.

Raw sequencing data were quality-checked with FASTQC (v0.11.5) and filtered using Trim-galore (v0.4.5) to obtain high-quality clean reads. The clean reads were aligned to the yellow catfish reference genome using TopHat (v2.1.1) [54] after index construction, generating BAM files for downstream analysis. Gene expression matrices were derived using HTSeq (v0.8.0) [55], and transcript abundances were estimated as FPKM (Fragments Per Kilobase of transcript per Million mapped fragments) with Cufflinks (v2.2.1) [56]. Differential expression analysis was performed in R (v4.3.3) with DESeq2 (v1.42.1) [57], retaining genes with mean FPKM > 1.0 in any group. Pairwise comparisons were conducted between infected, uninfected, and control groups for both skin and muscle tissues. Differentially expressed genes (DEGs) were identified using thresholds of *padj* < 0.05 and |log2FoldChange| ≥ 1.0. Functional enrichment analysis of DEGs was performed for Gene Ontology (GO) terms and Kyoto Encyclopedia of Genes and Genomes (KEGG) pathways using the DAVID database (https://davidbioinformatics.nih.gov/, accessed on 28 March 2024), with results visualized via the bioinformatics online platform (https://www.bioinformatics.com.cn/).

### 4.6. Integration Analysis of GWAS and RNA-Seq

In the GWAS analysis, the 100 kb upstream and downstream flanking regions of each associated SNP was regarded as a candidate interval. We annotated these candidate SNPs and performed pathway enrichment analysis. The overlapping region between immune-related genes and DEGs identified in skin and muscle tissues in the RNA-seq constitutes the genes under investigation in our study. Functional annotation of these overlapping genes was performed through pathway enrichment analysis to elucidate potential biological mechanisms underlying resistance to *V. mimicus* infection.

### 4.7. Validation

Total RNA from yellow catfish samples was extracted by TRIzol (Invitrogen, Carlsbad, CA, USA) according to the manufacturer’s instruction. RNA quantity and purity were determined by a NanoDrop2000 spectrophotometer (Thermo Fisher Scientific, Wilmington, DE, USA). Specifically, the absorbance ratio at 260 nm to 280 nm (A260/A280) was used as the key indicator of RNA purity, with values ranging from 1.8 to 2.1 considered acceptable (indicating minimal protein contamination and high RNA integrity). RNA integrity was further assessed by electrophoresis. Total RNA was diluted to 200 ng/μL with RNase-free deionized water. Subsequently, RNA was reverse transcribed into cDNA using PrimeScript™ RT reagent Kit with gDNA Eraser (Takara, Shanghai, China).

To elucidate the expression patterns of candidate genes identified through integrated GWAS and RNA-seq analyses, the expression levels of several related genes were evaluated by qRT-PCR. Primer designing for these genes was performed by using Primer Premier 5.0 and β-actin [11] was used as the internal control. The qRT-PCR reaction mixture consisted of 10 μL 2 × SYBR premix (Takara, Shanghai, China), 0.5 μL of each primer, 1 μL of cDNA template, and 7 μL of ddH_2_O. The qRT-PCR cycling condition was as follows: 95 °C for 3 min, 39 cycles at 95 °C for 10 s, 60 °C for 30 s, and 72 °C for 20 s. Melt curve analysis was performed by increasing the temperature from 65 °C to 95 °C, rising in 0.5 °C increment for 5 s. All reactions were performed in triplicates. Gene expression fold changes were determined according to the 2^−ΔΔCt^ method [58]. A t-test was performed using GraphPad Prism (v8.0.1) to determine the significance of gene expression differences between groups, with a statistical significance of *p* < 0.05. All the qRT-PCR primers were shown in Table 3. Gene-specific primers for *rac2*, *ncf1*, *ncf2*, *ncf4*, and the reference gene *β-actin* were designed based on reference sequences using NCBI BLAST (https://blast.ncbi.nlm.nih.gov/Blast.cgi, accessed on 25 March 2024) (primer sequences provided in Table 3).

### 4.8. Statistical Analysis

Quantitative data were analyzed using one-way ANOVA with GraphPad Prism (v8.0.1) to determine changes in expression levels of candidate genes following *V. mimicus* infection. Results are presented as mean ± standard error (mean ± SE). Statistical significance was determined at * *p* < 0.05, ** *p* < 0.01, and *** *p* < 0.001, with *p* > 0.05 considered non-significant. All analyses were performed with appropriate post hoc tests where applicable, maintaining α = 0.05 as the significance threshold.

## 5. Conclusions

In this study, we successfully established a natural *V. mimicus* infection model in yellow catfish based on a systematic elucidation of the characteristics of pathological damage in skin and muscle tissues and the regularity of immune responses. A synthesis of approaches involving genome-wide association studies and transcriptomic analyses was undertaken to identify key genetic loci and candidate genes associated with *V. mimicus* resistance. Through phenotypic screening and differential analysis, we identified a total of 6684 DEGs from skin and focused on 17 immunity-related candidate genes derived from GWAS. Integrated analysis revealed *rac2*, *yipf5*, *tmed7*, and *sgo1* as four significant genes. Among them, *rac2* was confirmed as a core functional gene involved in innate immune activation, particularly in neutrophil-mediated oxidative defense. Validation of gene expression confirmed the upregulation of *rac2* and its downstream effectors *ncf1* and *ncf4*, reinforcing its core role in host–pathogen defense. Additionally, pathway enrichment analysis of DEGs revealed that innate immune responses and programmed cell death were activated in skin tissue, while muscle tissue mainly redistributed metabolic resources to support immune protein synthesis. This study provides new insights into exploring the mechanisms of yellow catfish resistance to *V. mimicus* infection and screening resistance-related molecular markers, while laying a foundation for the development of disease-resistant aquaculture species.

## Figures and Tables

**Table 1:** Immune-related candidate genes in 500 kb genomic regions of yellow catfish.

Gene	Location	SNP Position	Type	*p-*Value
*rac2*	Chr15:3,214,559-3,231,661	3,227,652	Intronic (intron 5 of 6)	9.29 × 10^−9^
*yipf5*	Chr21:559,983-570,294	650,247	intergenic	9.83 × 10^−7^
*tmed7*	Chr14:13,634,530-13,646,992	13,668,655	intergenic	9.74 × 10^−7^
*sgo1*	Chr15:3,015,565-3,023,749	3,227,652	Intronic (intron 5 of 6)	9.29 × 10^−9^
*kif4*	Chr21:686,989-705,671	650,247	intergenic	9.83 × 10^−7^
*rnf14*	Chr21:313,328-323,403	650,247	intergenic	9.83 × 10^−7^
*brcc3*	Chr21:795,436-804,822	650,247	intergenic	9.83 × 10^−7^
*prdm1b*	Chr15:2,918,510-2,936,862	3,227,652	Intronic (intron 5 of 6)	9.29 × 10^−9^
*rnf121*	Chr21:666,310-686,058	650,247	intergenic	9.83 × 10^−7^
*gtpbp1*	Chr15:3,294,698-3,305,847	3,227,652	Intronic (intron 5 of 6)	9.29 × 10^−9^
*cdo1*	Chr14:13,650,644-13,660,086	13,668,655	intergenic	9.74 × 10^−7^
*commd10*	Chr14:13,679,409-13,719,550	13,668,655	intergenic	9.74 × 10^−7^
*pggt1b*	Chr14:13,592,033-13,611,778	13,668,655	intergenic	9.74 × 10^−7^
*sema6a*	Chr14:13,726,536-13,800,438	13,668,655	intergenic	9.74 × 10^−7^
*gtf2f1*	Chr15:3,425,989-3,435,323	3,227,652	Intronic (intron 5 of 6)	9.29 × 10^−9^
*stk26*	Chr21:192,041-212,970	650,247	intergenic	9.83 × 10^−7^
*pin1*	Chr18:19,547,172-19,553,617	19,797,963	intergenic	6.74 × 10^−7^

**Table 2:** Primers of PCR used for validation of HZAU2285.

Primer Name	Primer Sequence (5′-3′)
*V. mimicus-*F	CAGGTTTGYTGCACGGCGAAGA
*V. cholerae-*R	AGCAGCTTATGACCAATACGCC
*V. mimicus-*R	YCTTGAAGAAGCGGTTCGTGCA

**Table 3:** qRT-PCR primers for validation of differentially expressed genes between infected and control groups.

Gene	Forward Primer	Reverse Primer
*rac2*	TTGAGAACGTCAGAGCTAAATGGTA	TCTGATCGCTTCATCAAACACG
*ncf1*	GGCAGAGGAAAATCCAGAAGACA	TGGATGCTGACTCTCTTGCG
*ncf2*	CTCCAAAATGGTGCAGGTTGC	GTGCAACGCAGACGGATATG
*ncf4*	CTTGAACGCATGAGGGAGGT	GATGATGGATCTGGCGAGCA
*β-actin*	TGCTGCCTCTTCCTCCTCTC	GGACACCTGAACCTCTCATTGC

## Data Availability

The datasets generated from the current study are deposited in the NCBI Sequence Read Achieve (SRA) repository, under project number PRJNA1273481. The original contributions presented in this study are included in the article/Supplementary Materials. Further inquiries can be directed to the corresponding author.

## Supplementary Materials

The following supporting information can be downloaded at https://www.mdpi.com/article/10.3390/ijms27010441/s1.
