# Supplementary material for: Identification of Differentially Expressed Genes and SNPs Linked to Vibrio mimicus Resistance in Yellow Catfish (Pelteobagrus fulvidraco)
Source: Int J Mol Sci. 2025 Dec 31;27(1):441. doi: 10.3390/ijms27010441 (PMC12786896; doi:10.3390/ijms27010441)
Supplement: Supplementary file 1 [file ijms-27-00441-s001.zip › ijms-4033309-supplementary.pdf]

Supplementary Table S1. Correlation between growth traits (body length and body weight) and survival of yellow catfish in the infection experiment

Growth trait		Survival	Mortality
Length(cm)	Max	11.2	11.5
	Min	9.0	8.5
	Mean	10.1	9.9
	CV	6.7%	7.2%
	P-value	0.26	
Weight(g)	Max	29.2	30.6
	Min	11.9	11.6
	Mean	18.3	19.6
	CV	20.1%	21.0%
	P-value	0.06	

Supplementary Table S2. Summary of RNA-seq reads and the alignment rate

Groups	Sample ID	Raw reads	Clean reads	Alignment rate
**Skin(control)**	BO344-001T0001	416,865,16	40976290	76.10%
	BO344-001T0002	44,910,802	44,432,296	79.50%
	BO344-001T0003	47,310,072	46,793,024	78.90%
**Muscle(control)**	BO344-001T0004	38,258,940	38,230,410	74.30%
	BO344-001T0005	40,986,936	40,667,627	81.50%
	BO344-001T0006	41,108,732	40,816,680	85.10%
**Skin(infected)**	BO344-001T0007	39,829,206	39,747,336	75.40%
	BO344-001T0008	51,801,566	50,905,164	64.70%
	BO344-001T0009	44,593,282	44,471,008	71.00%
**Muscle(infected)**	BO344-001T00010	44,171,402	43,921,927	83.30%
	BO344-001T00011	42,684,096	42,413,008	83.70%
	BO344-001T00012	41,701,436	41,447,312	84.40%

A

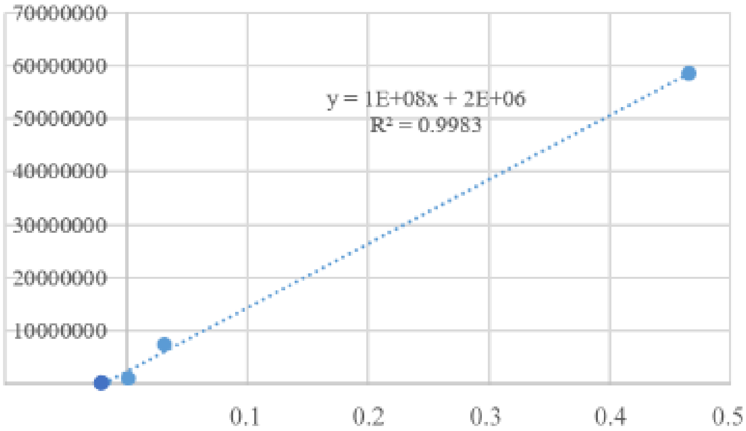

B

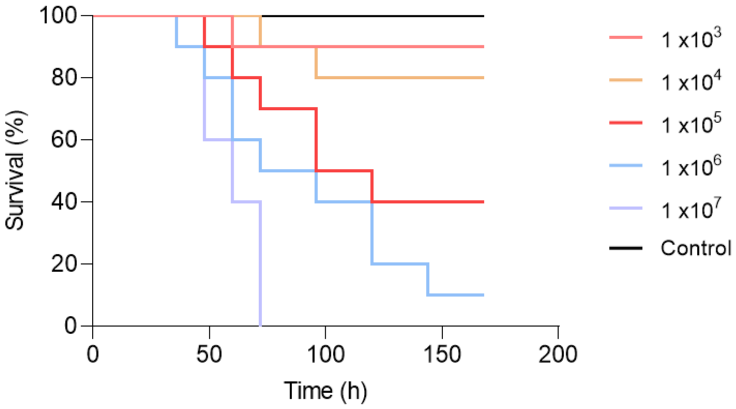

Supplementary Figure S1. Determination of the LC_{50} of *V.mimicus*. (**A**) Standard curve correlating bacterial concentration with absorbance; (**B**) Survival curves of yellow catfish after immersion treatment with different concentrations of bacterial solutions.

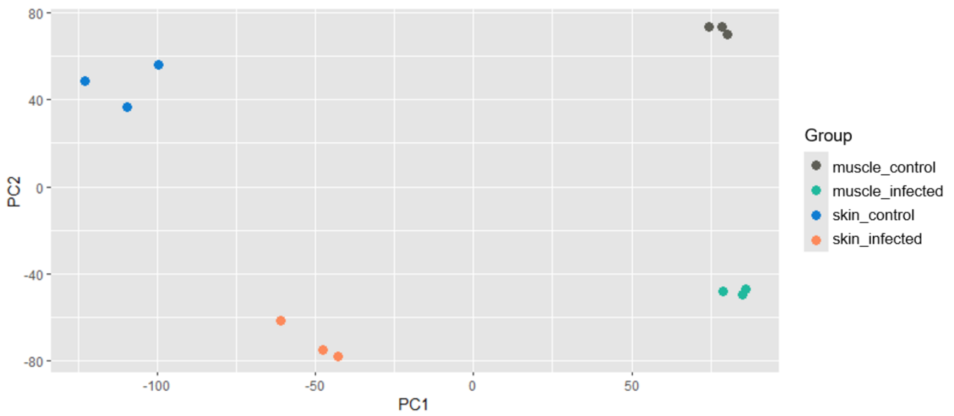

Supplementary Figure S2. PCA plot of skin and muscle samples from yellow catfish in transcriptome sequencing.

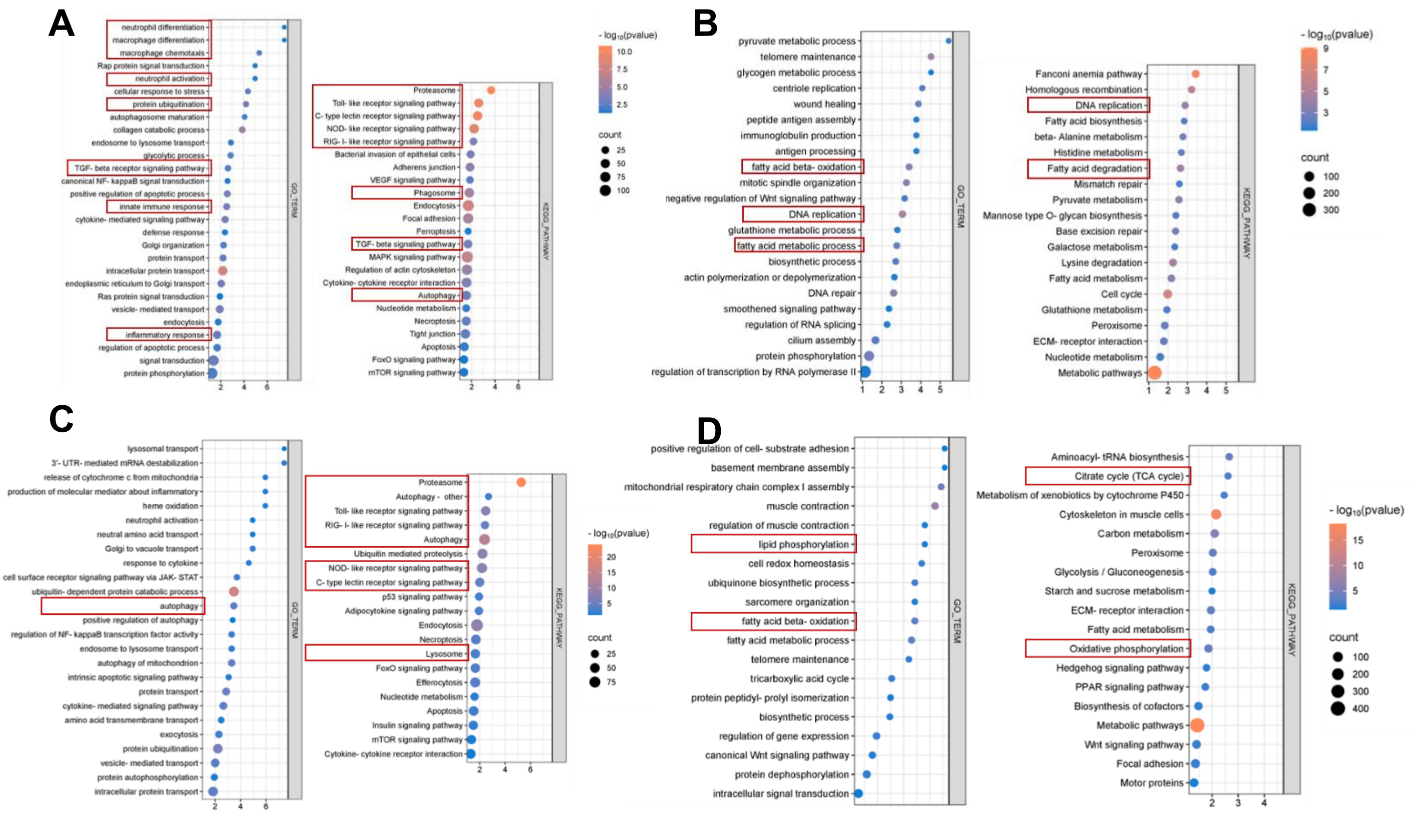

Supplementary Figure S3. Functional enrichment of DEGs. (**A**) Functional enrichment of up-regulated DEGs (infected vs. control) from skin; (**B**) Functional enrichment of down-regulated DEGs (infected vs. control) from skin; (**C**) Functional enrichment analysis of up-regulated DEGs (infected vs. control) from muscle; (**D**) Functional enrichment of down-regulated DEGs (infected vs. control) from muscle.
